# Two new species of *Rhaphium* from Qinghai Province, China (Diptera, Dolichopodidae, Rhaphiinae)

**DOI:** 10.3897/zookeys.931.49671

**Published:** 2020-04-30

**Authors:** Qilemoge, Chen Lin, Peifeng Qi, Deming Li, Ding Yang

**Affiliations:** 1 Department of Entomology, College of Plant Protection, China Agricultural University, Beijing 100193, China China Agricultural University Beijing China; 2 Institute of Life Science and Technology, Inner Mongolia Normal University, Huhhot, Inner Mongolia 010022, China Inner Mongolia Normal University Huhhot China; 3 Huzhu Beishan Forest, Haidong, Qinghai 810699, China Huzhu Beishan Forest Haidong China

**Keywords:** Long-legged fly, Palaearctic China, taxonomy, identification key

## Abstract

At present, there are 31 species of *Rhaphium* Meigen recorded in China. In this paper, two species from Qinghai Province of China are described as new to science: *Rhaphium huzhuense***sp. nov.**, *Rhaphium minhense***sp. nov.** A key to the Chinese species of *Rhaphium* is provided.

## Introduction

The genus *Rhaphium* Meigen belongs to the subfamily Rhaphiinae and contains 206 known species in the world (Yang et al. 2006; Yang et al. 2011; Grichanov 2017; Qilemoge et al. 2019; Grootaert 2019). Thirty-one species have been recorded in China, including 11 species distributed only in Oriental China, 17 species distributed only in Palaearctic China, two species from Oriental and Palaearctic China, and one species, *R. dilatatum* Wiedemann, 1830, with an unclear Chinese distribution (Yang et al. 2006; Qilemoge et al. 2019; Grootaert 2019).

The specimens upon which this study is based were collected in the Qinghai Province of China. The Qinghai Province is located in the northeastern part of the Tibetan Plateau in China, and has a continental climate. In this paper, we describe two new species of *Rhaphium* collected in this region. We provide an updated key to all Chinese species of *Rhaphium* with the exception of *R. dilatatum* and *R. relatus* (Becker, 1922), which are poorly described and lack known holotypes.

## Material and methods

The specimens in this study were collected in the forest by sweep nets and subsequently stored into 95% ethanol, and finally stored in the freezer (-20 °C). All specimens are deposited in the Entomological Museum of China Agricultural University (CAU), Beijing. Morphological terminology for adult structures mainly follows Cumming and Wood (2017). The following abbreviations are used: **acr** = acrostichal bristle(s), **ad** = anterodorsal bristle(s), **av** = anteroventral bristle(s), **cer** = cercus, **CuAx ratio** = length of m-cu / length of distal portion of CuA, **dc** = dorsocentral bristle(s), **npl** = notopleural bristle(s), **oc** = ocellar bristle(s), **pal** = postalar bristle(s), **pprn** = postpronotal bristle(s), **pvt** = postvertical bristle(s), **ial** = intra-alar bristle(s), **sc** = scutellar bristle(s), **sur** = surstylus, **vt** = vertical bristle(s).

## Taxonomy

### Rhaphium

Taxon classificationAnimaliaDipteraDolichopodidae

Meigen, 1803

F1FA3A96-AF25-59FE-9192-F32E83133B67

#### Diagnosis.

Body size small to large (1.5–5.7 mm); vertex flat; ocellar bristle nearly as long as vertical bristle; face narrower than frons; male clypeus not clearly separate from face; antenna black, first flagellomere mostly prolonged (2–10 times longer than wide), arista apical; propleuron with dense pale white hairs, without distinct bristle; vein M not bifurcated, R_4+5_ parallel or slightly convergent with M apically, CuAx ratio less than 1; abdominal segments 1–3 usually with long pale hairs, abdominal segment 6 visible and pubescent; male genitalia connected tightly with pregenital segment, cap-like; epandrium wide apically, epandrial lobe generally simple with bristle; surstylus bifurcate or not; cercus varied, usually long and narrowed towards tip, sometimes bifurcate, with hairs and bristles at middle; hypandrium simple (Yang et al. 2011).

### Key to species (males) of *Rhaphium* from China

**Table:** 

1	First flagellomere at least 4.0 times longer than wide	**2**
–	First flagellomere at most 2.5 times longer than wide	**17**
2	Four dc	**3**
–	Five to six dc	**6**
3	First flagellomere less than 5.0 times longer than wide; acr present	**4**
–	First flagellomere more than 7.5 times longer than wide; acr absent	***R. sichuanense* Yang & Saigusa, 1999**
4	All coxae black; femora mostly black	***Rhaphium minhense* sp. nov.**
–	At least fore coxa yellow, femora mostly yellow	**5**
5	All coxae yellow	***R. apicinigrum* Yang & Saigusa, 1999**
–	Only fore coxa yellow	***R. huzhuense* sp. nov.**
6	Arista apically inflated (Yang et al. 2011: fig. 809)	***R. parentianum* Negrobov, 1979**
–	Arista simple, not inflated at apex	**7**
7	Cercus bifurcate	**8**
–	Cercus not bifurcate	**10**
8	First flagellomere at most 7.0 times longer than wide	**9**
–	First flagellomere at least 9.0 times longer than wide	***R. bilobum* Tang, Wang & Yang, 2016**
9	All coxae black	***R. shaliuhense* Qilemoge, Wang & Yang, 2019**
–	All coxae yellow	***R. daqinggouense* Tang, Wang & Yang, 2016**
10	First flagellomere at least 8.0 times longer than wide	**11**
–	First flagellomere at most 6.0 times longer than wide	**12**
11	Eight uniseriate acr; cercus nearly triangular, short, not bifurcated	***R. neimengense* Tang, Wang & Yang, 2016**
–	Five to eight irregularly paired acr; cercus deeply bifurcated into 2 long lobes (Yang et al. 2011: fig. 816)	***R. zhongdianum* Yang & Saigusa, 2001a**
12	Surstylus bifurcate apically	**13**
–	Surstylus simple	**14**
13	Acr absent; all coxae yellow	***R. furcatum* Yang & Saigusa, 2000**
–	Acr present; fore coxa brown at base, mid and hind coxae brown with yellow apex	***R. spinulatum* Grootaert, Taylor & Guénard, 2019**
14	Five dc; surstylus without apical incision	**15**
–	Six dc; surstylus with apical incision	**16**
15	Hind coxa yellow; surstylus thick, apically straight	***R. palliaristatum* Yang & Saigusa, 2001b**
–	Hind coxa brown with yellow apex; surstylus thin, apically rounded	***R. hongkongense* Grootaert, Taylor & Guénard, 2019**
16	All coxae yellow; hind tibia yellow; surstylus with long thick hairs apically; cercus long ribbon-like (Yang et al. 2011: fig. 815)	***R. xinjiangense* Yang, 1998a**
–	Only fore coxa yellow, mid and hind coxae black; hind tibia black; surstylus only with sparse short hairs; cercus elongate triangular (Yang et al. 2011: fig. 810)	***R. qinghaiense* Yang, 1998b**
17	Fore tarsus modified (inflated, depressed or with Y-shaped bristle)	**18**
–	Fore tarsus simple	**23**
18	Fore tarsomere 1 simple, fore tarsomere 5 with 2 Y-shaped apical bristles and 2 long strong bristles	***R. dorsiseta* Tang, Wang & Yang, 2016**
–	Fore tarsomere 1 modified	**19**
19	Fore tarsomere 1 depressed dorsally but strongly raised ventrally	***R. lumbricus* Wei, 2006**
–	Fore tarsomere 1 inflated apically	**20**
20	Arista distinctly (1.4×) longer than first flagellomere	**21**
–	Arista nearly as long (0.8×) as first flagellomere (Yang et al. 2011: fig. 813)	***R. sinense* Negrobov, 1979**
21	Fore tarsomere 2, and mid tarsomeres 4 and 5 inflated (Yang et al. 2011: fig. 800)	***R. baihuashanum* Yang, 1998a**
–	Fore and mid tarsi simple, not inflated	**22**
22	Middle and lower postocular bristles yellow; 8 dc; mid femur yellow; cercus not bifurcate, narrowed toward apex	***R. heilongjiangense* Wang, Yang & Masunaga, 2005**
–	All postocular bristles black; 5 dc; mid femur black; cercus bifurcate	***R. gangchanum* Qilemoge, Wang & Yang, 2019**
23	Fore femur with row of strong ventral bristles or long ventral hairs	**24**
–	Fore femur without row of distinct ventral bristles or hairs	**25**
24	First flagellomere about 2.0 times as long as wide; arista about 2.0 times longer than first flagellomere; fore femur with row of long pale yellow ventral bristles as long as width of fore femur; cercus narrowed at base and widened towards apex, with distinct marginal denticles (Yang et al. 2011: fig. 811)	***R. riparium* (Meigen, 1824)**
–	First flagellomere about 1.5 times as long as wide; arista nearly 3.0 times longer than first flagellomere; fore femur with 2 rows of long pale yellow bristles longer than width of fore femur; cercus very long, wide in basal half	***R. apophysatum* Tang, Wang & Yang, 2016**
25	Fore tarsus modified, tarsomere 1 with row of strong ventral bristles on basal half, tarsomere 2 inflated apically (Yang et al. 2011: fig. 807c)	**26**
–	Fore tarsus simple, tarsomere 1 without distinct ventral bristles, tarsomere 2 simple	**27**
26	Fore and mid femora yellow apically, fore and mid tibia yellow; fore coxa with black bristles and hairs	***R. micans* (Meigen, 1824)**
–	Fore femur, mid and hind tarsi dark; fore coxa with light yellow bristles and hairs	***R. dispar* Coquillett, 1898**
27	All coxae dark, fore and mid femora yellow apically	**28**
–	Basal half of fore coxa and apical 1/3 of hind femur dark	**29**
28	Hind tibia with 3 ventral bristles; mid tarsomere 1 about 1.1 times as long as hind tarsomere 1	***R. wuduanum* Wang, Yang & Masunaga, 2005**
–	Hind tibia without distinct ventral bristles; mid tarsomere 1 about 1.5 times as long as hind tarsomere 1	***R. gansuanum* Yang, 1998a**
29	Mid coxa with 1 strong outer bristle, and bunch of ventral bristles; mid tibia with 1 av	**30**
–	Mid coxa only with only 1 strong outer bristle at middle, without bunch of ventral bristles; mid tibia without ventral bristles	***R. bisectum* Tang, Wang & Yang, 2016**
30	Calypteral fringe with yellow hairs; cercus not bifurcate; surstylus short and thick	**31**
–	Calypteral fringe with black hairs; cercus bifurcate; surstylus basally thick, apically sharp, with one protuberance	***R. tianshuiense* Qilemoge, Wang & Yang, 2019**
31	First flagellomere less than 2.0 times as long as wide; fore femur entirely yellow	***R. canniccii* Grootaert, Taylor & Guénard, 2019**
–	First flagellomere 2.0 times as long as wide; fore femur brownish except yellow at apex	**32**
32	Arista only slightly longer (1.2×) than first flagellomere	***R. mediocre* (Becker, 1922)**
–	Arista 3.0 times as long as first flagellomere	***R. eburnean* (Parent, 1926)**

### Rhaphium huzhuense sp. nov.

Taxon classificationAnimaliaDipteraDolichopodidae

C9D0A460-E24D-54CF-9E9E-E9BF743D8232

http://zoobank.org/4D9C6328-169D-4CD3-8450-B50C9C01EB35

Figs 1 , 3 , 4

#### Diagnosis.

First flagellomere 7.5 times longer than wide. Fore coxa yellow, mid and hind coxae black except for yellow tip; hind femur black dorsally near apex; all tibiae yellow. CuAx ratio 0.35. Calypteral fringe with yellow hairs. Surstylus triangular, apically with four strong bristles. Cercus bifurcate, outer lobe long, slender, curved; inner lobe strip-like, apically with three strong bristles.

#### Description.

**Figures 1, 2.**
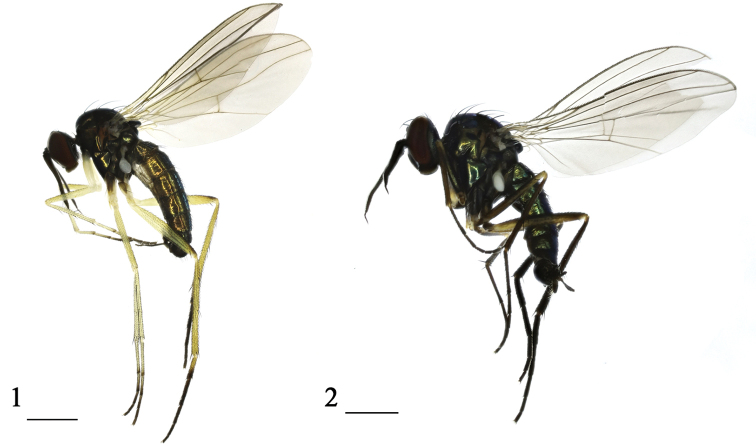
Habitus, lateral view **1***Rhaphium huzhuense* sp. nov., holotype male **2***Rhaphium minhense* sp. nov., holotype male. Scale bars: 1 mm.

**Male** (Fig. 1). ***Body*** length 3.6–3.75 mm. ***Wing*** length 4.0–4.2 mm.

**Figures 3–6.**
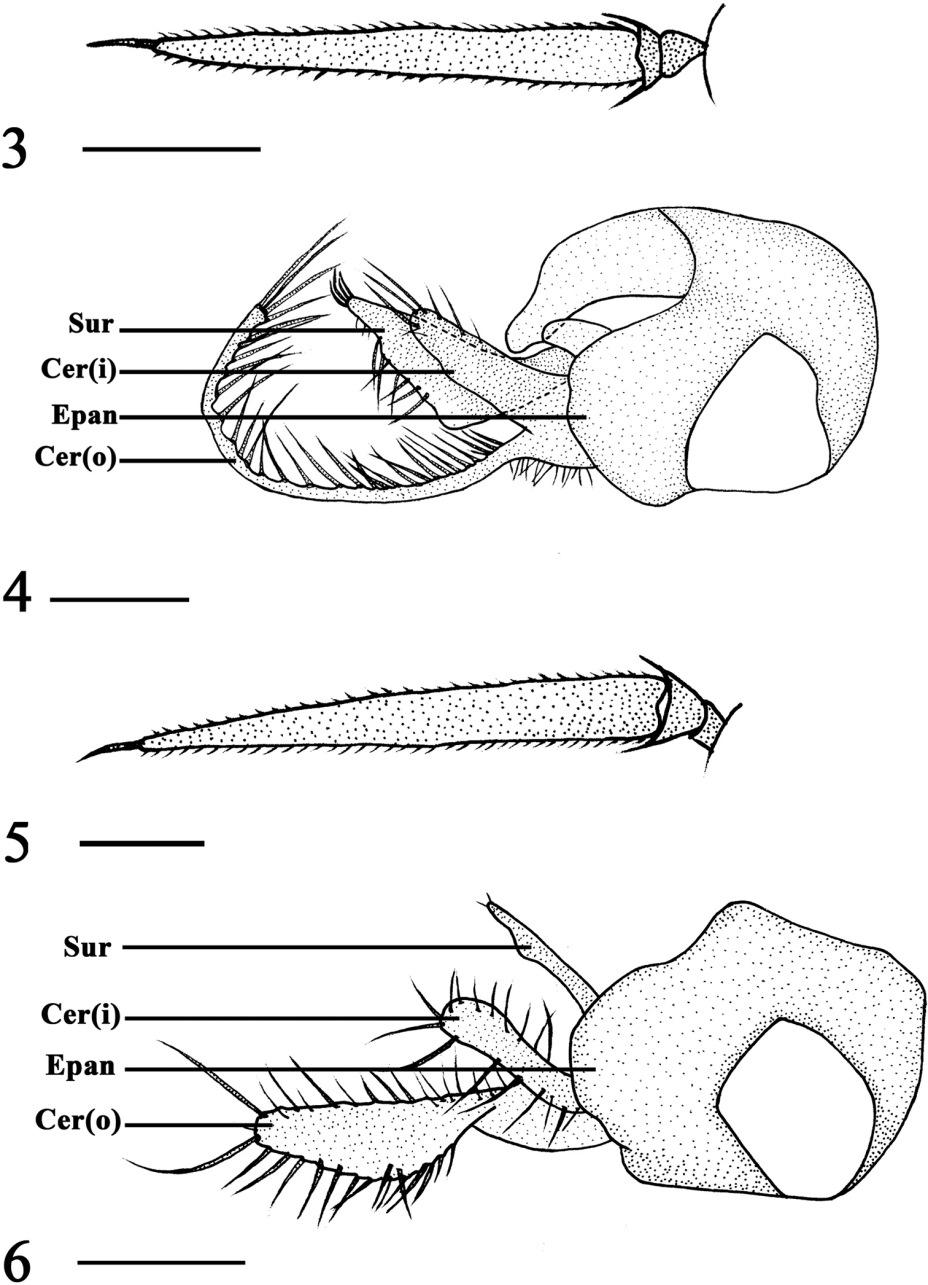
*Rhaphium huzhuense* sp. nov., male **3** antenna, lateral view **4** genitalia, lateral view. *Rhaphium minhense* sp. nov., male **5** antenna, lateral view **6** genitalia, lateral view. Abbreviations: sur = surstylus, cer (o) = outer lobe of cercus, cer (i) = inner lobe of cercus, epan = epandrium. Scale bars: 0.2 mm.

***Head*** metallic green with pale gray pruinescence. Face black with pale gray pruinescence. Frons brown with pale gray pruinescence. Upper postocular bristles black, middle and lower postocular bristles yellow. Two oc, two vt, two pvt. Antenna (Fig. 3) black; scape bare; pedicel with hairs; first flagellomere elongated, 7.5 times longer than wide, apically sharp; arista black, inserted at apex, basal aristomere 1/4 as long as apical aristomere. Proboscis and palpus black with yellow hairs.

***Thorax*** metallic green with pale gray pruinescence. Hairs and bristles on thorax black. Four strong dc, four irregular pairs of acr, two strong npl, one strong sutural ial, two strong pa, one strong anterior pprn; scutellum with one pair of sc. Legs yellow, except for basal part of mid and hind coxae black; hind femur black dorsally near apex; fore and mid tarsi from tip of tarsomere 2 onwards black, tip of tarsomere 1 of fore and mid leg black, hind tarsus from tip of tarsomere 1 onward black. Most hairs and bristles on legs black, fore coxa with yellow bristles, and mid and hind coxae each with one black outer bristle. All femora without ventral bristles, mid and hind femora each with one black preapical bristle. Fore tibia with one ad, two pv, middle with one av, and two apical bristles; mid tibia with two ad, one pd, basal half with one av, and three apical bristles; hind tibia with two ad, two pd, three av (basal half with one av, apical half with two av), and three apical bristles. Relative lengths of femur, tibia and 5 tarsomeres, fore leg 2.6 : 2.8 : 1.3 : 0.8 : 0.6 : 0.4 : 0.4; mid leg 4.0 : 4.3 : 1.9 : 1.2 : 0.8 : 0.6 : 0.4; hind leg 4.7 : 5.2 : 1.3 : 1.5 : 1.0 : 0.7 : 0.5. Wing hyaline, veins black; M bent medially, M and R_4+5_ parallel apically; CuAx ratio 0.35. Calypteral fringe yellow with yellow hairs. Halter yellow.

***Abdomen*** metallic green with pale gray pruinescence with hairs and bristles black. Male genitalia (Fig. 4): epandrium black, nearly as long as wide. Epandrial lobe short, rounded apically, without distinct bristle. Surstylus on epandrium black, nearly triangular, outside margin with seven strong bristles and apex with four strong bristles. Cercus black, bifurcate, outer lobe long, slender, curved with strong bristles along length ventrally; inner lobe wider and shorter, strip-like, apically with three strong bristles.

**Female.** Unknown.

#### Types.

**Figures 7, 8.**
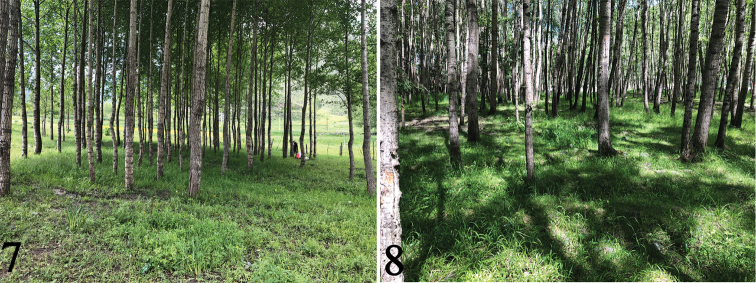
Habitat **7** Qinghai, Minhe, Tangeryuan Forest **8** Qinghai, Huzhu, Songduo Forest.

***Holotype*** male, China, Qinghai, Huzhu, Songduo Forest, 3165 m; 2019.VII.1, leg. Qilemoge (CAU), collected by sweep nets in grassland. ***Paratypes***: two males, same data as holotype; six males, China, Qinghai, Minhe, Tangeryuan Forest, 2304 m, 2019.VI.28, leg. Xin Li (CAU), collected by sweep nets in grassland. (Figs 7, 8).

#### Distribution.

Palaearctic: China (Qinghai).

#### Remarks.

The new species is similar to *R. apicinigrum* Yang & Saigusa, 1999, but these two species can be separated by several features. In *R. huzhuense*, the first flagellomere is about 7.5 times longer than wide (Fig. 3); the fore coxa is yellow, the mid and hind coxae are black with the yellow apex; the hind femur is black apico-dorsally; and the surstylus is nearly triangular, not bifurcated (Fig. 4). In *R. apicinigrum*, the first flagellomere is about 4.3 times longer than wide; all coxae are yellow; and the surstylus is long and apically bifurcated (Yang et al. 2011: 1248, fig. 799a, b).

#### Etymology.

The specific name refers to the type locality, Huzhu.

### Rhaphium minhense sp. nov.

Taxon classificationAnimaliaDipteraDolichopodidae

FFFCB251-BD63-56ED-98FB-FEF4743F6DF8

http://zoobank.org/B8FD1531-B5DD-4D83-AE5A-64B47886FD90

Figs 2 , 5 , 6

#### Diagnosis.

First flagellomere 6.5 times longer than wide. Legs mostly black except fore and mid femora ventrally yellow at tip and hind femur ventrally yellow on basal 3/4. CuAx ratio 0.36. Calypteral fringe with black hairs. Surstylus finger-like, ventrally with one protuberance. Cercus bifurcate, outer lobe twisted at middle, apical half nearly triangular; inner lobe strip-like with strong bristles.

#### Description.

**Male** (Fig. 2). ***Body*** length 4.0 mm. ***Wing*** length 4.6 mm.

***Head*** metallic green with pale gray pruinescence. Face dark metallic green with silvery white pruinescence. Frons brown with pale gray pruinescence. Upper postocular bristles black, middle and lower postocular bristles yellow. Two oc, two vt, two pvt. Antenna (Fig. 5) black; scape bare; pedicel with hairs; first flagellomere elongated, 6.5 times longer than wide, with acute apex; arista black, inserted at apex, basal aristomere 1/4 as long as apical aristomere. Proboscis black with yellow hairs, palpus black with black hairs.

***Thorax*** metallic green with pale gray pruinescence. Hairs and bristles on thorax black. Four strong dc, four irregular pairs of acr, two strong npl, one strong sutural ial, two strong pa, one strong anterior pprn; scutellum with one pair of sc. Legs black, except for fore and mid femora ventrally yellow at tip, hind femur ventrally yellow at basal 3/4. Most hairs and bristles on legs black. Fore and mid coxae with yellow bristles, hind coxa with one black outer bristle. Mid and hind femora each with one black preapical bristle. Fore tibia with one ad, one pd, basal half with two av, and two apical bristles; mid tibia with two ad, one pd, apical half with one av, and four apical bristles; hind tibia with two ad, two pd, three av (middle with one av, apical half with two av), and four apical bristles. Relative lengths of femur, tibia and 5 tarsomeres, fore leg 2.4 : 2.5 : 1.2 : 0.5 : 0.4 : 0.2 : 0.3; mid leg 3.1 : 3.0 : 1.6 : 0.7 : 0.5 : 0.4 : 0.4; hind leg 3.5 : 3.9 : 1.6 : 1.6 : 0.8 : 0.5 : 0.4. Wing hyaline, veins black; M bent medially, M and R_4+5_ parallel apically; CuAx ratio 0.36. Calypteral fringe yellow with black hairs. Halter yellow.

***Abdomen*** metallic green with pale gray pruinescence with hairs and bristles black. Male genitalia (Fig. 6): epandrium black, nearly as long as wide. Surstylus on epandrium black, thin, finger-like, apically sharp, with two weak bristles, ventrally with one protuberance. Cercus bifurcate, outer lobe thick, twisted at middle, nearly triangular at apical half, apically with two long strong bristles; inner lobe strip-like, apically rounded, with three strong bristles, ventrally with five long strong bristles.

**Female.** Unknown.

#### Types.

***Holotype*** male, China, Qinghai, Minhe, Tangeryuan Forest, 2304 m, collected by sweep nets in grassland, 2019.VI.28, leg. Xin Li (CAU) (Fig. 8).

#### Distribution.

Palaearctic: China (Qinghai).

#### Remarks.

The new species is similar to *R. shaliuhense*Qilemoge et al., 2019, but both species can be separated by several features. In *R. minhense*, the body length is 4.0 mm; the thorax has four strong dc; the fore and mid femora are yellow ventrally at tip; the Calypteral fringe has black hairs; the outer lobe of cercus is twisted at middle, the apical half part is nearly triangular (Fig. 6). In *R. shaliuhense*, the body length is 2.5 mm; the thorax has five strong dc; the fore and mid femora are black; the Calypteral fringe has yellow hairs; the outer lobe of the cercus is strip-like (Qilemoge et al. 2019: 94, fig. 7).

#### Etymology.

The specific name refers to the type locality, Minhe.

## Discussion

*Rhaphium* is the largest genus in Rhaphiinae and including the species described here there are now 33 species documented to occur in China. Several species groups have been proposed within *Rhaphium*, for example Negrobov (1986) proposed a key to Palaearctic and Nearctic species of the *R. nasutum* group, having the following characters: hind coxa with a group of lateral hairs, fore tarsomere 1 with a row of short black bristles, cercus divided into lobes. Grichanov (2004) and Naglis (2009) mentioned the *R. albifrons* group, with the following combination of characters: hind coxa with a strong white lateral bristle; fore tarsomere 1 without comb of strong bristles; a key to males was provided. Negrobov and Grichanov (2010) described, and provided a key to, the *R. crassipes* group, diagnosed by: mid tarsomeres 4–5 black, dilated and flattened dorsoventrally. Naglis and Grootaert (2011) published the *R. srilankensis* group, with the sole nominal species notable for the arista being absent in males, and provided a key to Oriental genera of Rhaphiinae. Negrobov et al. (2011) proposed the *R. tridactylum* group, included four species, and provided a key to species. Negrobov et al. (2013) described the *R. ensicorne* group in which the cercus is bifoliate. Tang et al. (2016) mentioned the *R. bilobum* group with the defining characters: thorax with 5 dc; cercus bifurcate with two simple lobes and the *R. flavilabre* group, diagnosed by: thorax with 5 dc; male genitalia shorter than epandrium, with long pale apical bristles which are at least as long as epandrium. Grootaert et al. (2019) proposed the *R. micans* group, which differed by the following characters: cercus long, flattened, nearly twice as long as epandrium. The two new species described here do not fit the diagnoses of any of the above species groups.

Previously, there were 31 species recorded from China. Here we report two new species of *Rhaphium* from the Qinghai Province of Palaearctic China. *Rhaphium* can be considered a widespread genus in China. However, Ningxia, Xinjiang and Shanxi have few species, which might be due to the relatively dry climates of these provinces. The sole species (*R. heilongjiangense*) was known from the northeastern provinces (Heilongjiang, Jilin, Liaoning) of China: inadequate collection might be another reason for lower species diversity (Yang et al. 2011).

## Supplementary Material

XML Treatment for Rhaphium

XML Treatment for Rhaphium huzhuense

XML Treatment for Rhaphium minhense

## References

[B1] Becker T (1922) Dipterologische Studien, Dolichopodidae der indo-australischen Region. Capita Zoologica 1(4): 1–247. 10.5962/bhl.title.132893

[B2] Cumming JM Wood DM (2017) Adult Morphology and Terminology. In: Kirk-Spriggs AH Sinclair BJ (Eds) Manual of Afrotropical Diptera (Vol.1). South Africa National Biodiversity Institute, Pretoria, 89–133.

[B3] Grichanov IY (2004) A list of Dolichopodidae from the Tyresta National Park, Sweden, with description of a new species of the genus *Rhaphium* Meigen (Diptera). Zoosystematica Rossica 12(2): 267–269.

[B4] Grichanov IY (2017) Alphabetic list of generic and specific names of predatory flies of the epifamily Dolichopodidae (Diptera) (2^nd^ ed.). Plant Protection News Supplements 23: 442–455.

[B5] Grootaert P Taylor C Guénard B (2019) Three new species of *Rhaphium* Meigen, 1803 from mangroves in Hong Kong (Diptera: Dolichopodidae: Rhaphiinae). European Journal of Taxonomy 540: 1–21. 10.5852/ejt.2019.540

[B6] Naglis S (2009) Some taxonomical changes in the genus *Rhaphium* (Diptera, Dolichopodidae), with a key to the *Rhaphium albifrons* species group. Mitteilungen der Schweizerischen Entomologischen Gesellschaft 82: 201–203. 10.11646/zootaxa.2991.1.6

[B7] Naglis S Grootaert P (2011) A remarkable new species of *Rhaphium* Meigen (Diptera, Dolichopodidae) from Sri Lanka. Zootaxa 2991: 44–48.

[B8] Negrobov OP (1979) Dolichopodidae. In: Lindner E (Ed.) Die Fliegen der Palaearktischen Region 4(5).Lieferung 322: 475–530.

[B9] Negrobov OP (1986) Holarctic links in family Dolichopodidae. Biogeografiya Beringiiskogo sektora Subarktiki: Materialy Vsesoyuz, Simpoz, Magadan 1983: 161–168.

[B10] Negrobov OP Barkalov AV Selivanova OB (2011) New dolichopodid species of the genera *Dolichopus Latreille*, 1797 and *Rhaphium Meigen*, 1803 (Diptera, Dolichopodidae) from Siberia. Euroasian Entomological Journal 10(2): 203–206.

[B11] Negrobov OP Grichanov IY (2010) The *Rhaphium crassipes* species group in the Palearctic Region with the description of a new species from Uzbekistan (Diptera: Dolichopodidae). Caucasian Entomological Bulletin 6(1): 117–122. 10.23885/1814-3326-2010-6-1-117-122

[B12] Negrobov OP Grichanov IY Selivanova OV (2013) Palearctic species of the *Rhaphium albifrons* group (Diptera, Dolichopodidae). Euroasian Entomological Journal 12(6): 601–606.

[B13] Parent O (1926) Dolichopodidés nouveaux de l’extrême orient paléarctique. Encyclopédie entomologique. Serie B. II. Diptera (Vol. 3). Paris, 111–149.

[B14] Qilemoge Wang MQ Yang D (2019) Three new species of *Rhaphium* from China, with an updated key to Chinese *Rhaphium* (Diptera, Dolichopodidae, Rhaphiinae). Zookeys 840: 84–99. 10.3897/zookeys.840.31602 PMC6482115 31065228

[B15] Tang CF Wang N Yang D (2016) *Rhaphium* (Diptera: Dolichopodidae: Rhaphiinae) from China with six new species. Zootaxa 4162(3): 581–593. 10.11646/zootaxa.4162.3.11 27615993

[B16] Wang MQ Yang D Masunaga K (2005) Notes on *Rhaphium* Meigen from the Chinese mainland (Dilichopodidae, Diptera). Transactions of the American Entomological Society 131: 403–409.

[B17] Wei L (2006) Dolichopodidae. In: Li Z Jin D (Eds) Insects from Fanjingshan Landscape.Guizhou Science and Technology Publishing House, Guiyang, 468–502.

[B18] Yang D (1998a) New and little known species of Dolichopodidae from China (I). Bulletin de l’ Institute Royal des Sciences Naturelles de Belgique, Entomologie 68: 151–164.

[B19] Yang D (1998b) New and little known species of Dolichopodidae from China (III). Bulletin de l’ Institute Royal des Sciences Naturelles de Belgique, Entomologie 68: 177–183.

[B20] Yang D Saigusa T (2001) New and little known species of Dolichopodidae (Diptera) from China (IX). Bulletin de l’Institut Royal des Sciences Naturelles de Belgique Entomologie 71, 165–188.

[B21] Yang D Saigusa T (1999) New and little known species of Dolichopodidae from China (VI). Bulletin de l’ Institute Royal des Sciences Naturelles de Belgique, Entomologie 69: 233–250.

[B22] Yang D Saigusa T (2000) New and little known species of Dolichopodidae from China (VII): Diptera from Emei Mountain (2). Bulletin de l’ Institute Royal des Sciences Naturelles de Belgique, Entomologie 70: 219–242.

[B23] Yang D Saigusa T (2001a) New and little known species of Dolichopodidae (Diptera) from China (IX). Bulletin de l’ Institute Royal des Sciences Naturelles de Belgique, Entomologie 71: 165–188.

[B24] Yang D Saigusa T (2001b) New and little known species of Dolichopodidae (Diptera) from China (XI). Bulletin de l’ Institute Royal des Sciences Naturelles de Belgique, Entomologie 71: 237–256.

[B25] Yang D Zhang LL Wang MQ Zhu YJ (2011) Fauna Sinica Insecta (Vol. 53). DipteraDolichopodidae. Science Press, Beijing, 1241–1273.

[B26] Yang D Zhu YJ Wang MQ Zhang LL (2006) World catalog of Dolichopodidae (Insecta: Diptera). China Agricultural University Press, Beijing, 340–357.

